# Mass Tracking in Cellular Networks for the COVID-19 Pandemic Monitoring

**DOI:** 10.3390/s21103424

**Published:** 2021-05-14

**Authors:** Emil J. Khatib, María Jesús Perles Roselló, Jesús Miranda-Páez, Victoriano Giralt, Raquel Barco

**Affiliations:** 1 Department of Communications Engineering, Universidad de Málaga, 29071 Málaga, Spain; rbarco@uma.es; 2 Department of Geography, Universidad de Málaga, 29071 Málaga, Spain; mjperles@uma.es; 3 Department of Psychobiology and Methodology of Behavioral Sciences, Universidad de Málaga, 29071 Málaga, Spain; jmpaez@uma.es; 4 Digital Transformation Vicerectorate, Universidad de Málaga, Innovation Director, 29071 Málaga, Spain; victoriano@uma.es

**Keywords:** location, cellular networks, COVID-19, pandemic

## Abstract

The year 2020 was marked by the emergence of the COVID-19 pandemic. After months of uncontrolled spread worldwide, a clear conclusion is that controlling the mobility of the general population can slow down the propagation of the pandemic. Tracking the location of the population enables better use of mobility limitation policies and the prediction of potential hotspots, as well as improved alert services to individuals that may have been exposed to the virus. With mobility in their core functionality and a high degree of penetration of mobile devices within the general population, cellular networks are an invaluable asset for this purpose. This paper shows an overview of the possibilities offered by cellular networks for the massive tacking of the population at different levels. The major privacy concerns are also reviewed and a specific use case is shown, correlating mobility and number of cases in the province of Málaga (Spain).

## 1. Introduction

The COVID-19 pandemic, which is still raging at the moment of writing this paper, has caused 123.15 million infections and 2.71 million deaths [1]. This, in itself, makes the COVID-19 pandemic the worst single global-scale disaster of the 21st century so far. However, the legacy of COVID-19 is multifaceted and complex; it has shown the fragility of the post-industrial society when confronting an invisible and unexpected enemy [2]. There were virtually no measures to prevent the virus from spreading worldwide even when the hazard was well known to everyone, and there was an inability for detecting cases in time to prevent further contagion. Even at the time of counting the fatalities, the data collection systems have shown major discrepancies [3], evidencing a total lack of basic data management strategies. Moreover, the very fact that the pandemic was unexpected, despite warnings by some studies [4], shows the unpreparedness of public administrations and society in general.

Mobility is the key ingredient for transforming an epidemic into a pandemic, since people unknowingly carry the virus from an infected community into a clean one that is geographically isolated. The importance of mobility is obvious when studying, for instance, the end of the 15th and the 16th century [5], when the Spanish explorers arrived at America, establishing contact between two civilizations that had been isolated for thousands of years and exposing American natives for the first time to viruses such as smallpox and measles and Europeans to syphilis. Fast-forward to the 21st century, and a hyper-connected transport network allows people (and viruses) to cross the world in a matter of hours. It is very difficult for any single community not to be in contact with any other community of the whole world through a human chain of a few hops [6]. A virus would only need to be easily transmissible to become a pandemic in a margin of days or weeks [7]. Therefore, efficient monitoring of mobility is a must if any kind of control or contention mechanism is to be used; or, at the very least, it can provide a vital head start for the preparation of palliative actions.

There are diverse solutions for tracking human mobility: public transportation usage monitoring [8], aggregation of ticketing information [9], credit card transaction analytics, etc. These technologies, combined with Big (Geo) Data analytics have proven useful for the analysis of population flow [10].

However, if there is one single technology in recent years that has the potential of knowing where people are, and where they go, that is cellular network-based location [11]. Inherently, mobile networks need and obtain information of the location of each individual for their operation, with different degrees of precision depending on the application and the privacy policies. They are, therefore, an invaluable data source, given their high dissemination among the global population, ease for data gathering, and ability to do so in real-time. The major debate, which is still open today, concerns the privacy concerns and the potential that it also has for becoming a mass surveillance tool [12].

This paper explores the potential of mobile networks for tracking movements of people and relating them to the spread of COVID-19 and any other pandemic at different levels (from individual user monitoring to massive flows of population). A detailed review of the underlying technologies is done, and the privacy concerns and possible solutions are described. The different possibilities offered for pandemic control are outlined, and finally, a sample use case with real contagion and mobility data is shown as a proof of concept of the technology and its potential.

In Section 2, the aspects of human mobility and virus spreading are explored in more detail. In Section 3, the existing technologies for location are described, and the privacy concerns are listed in Section 4. The application of cellular network location for pandemics is then described in Section 5. In Section 6, the materials for the use case analysis are described and in Section 7 the analysis results are shown and discussed. Finally, the conclusions are reviewed in Section 8.

## 2. Human Mobility and COVID-19 Spreading

COVID-19 is an airborne virus [13], meaning it can be transmitted by the air through the respiratory system without direct contact between infected persons (called patient if they have symptoms, or carriers if they are asymptomatic) and potential new victims (exposed individuals). Therefore, proximity between people for long periods of time in closed spaces and crowds favors transmission. The presence of closed spaces and crowd formation frequency are features of specific geographical units. For instance, zones with a high number of shops will tend to concentrate people in closed spaces and are prone to crowd build-ups, especially on dates such as near Christmas or during sales. This will influence the number of infections in the inhabitants or bypassers of that zone.

To alleviate the effects of proximity, in the last year, social distancing measures have been recommended to the population, resulting in varying behavioral changes in different countries, as reviewed in [14]. While this is an effective measure, it is not always possible to keep social distancing in closed spaces, such as in public transportation [15]. To reduce the risk, limitation of capacity has been enforced in closed spaces along with recommendations of keeping spaces ventilated.

In communities with a high population density (mainly located in cities [16]), where people share common spaces such as buses or lifts, the virus spreads rapidly and easily, giving rise to *hotspots*. The virus then travels from hotspot to hotspot with social interaction between members of different communities.

As a consequence, public authorities have implemented diverse mobility limitation strategies:

- Geographical unit isolation: traffic between geographical units (e.g., a region, province, or even Sub-City Districts or SCDs) has been forbidden to keep the virus contained and protect regions with a lower incidence. The example of the regional isolation in Italy during the second wave is studied in [17].
- Lockdowns: either at a national or local scale, citizens were mandated to stay isolated at home except for basic necessities to prevent interactions that can lead to contagion. Reference [18] studies the effects of the worldwide lockdowns during the first wave up to May 2020.
- Curfews: similar to lockdowns, curfews limit freedom of movement at certain hours of the day, under the premise that certain leisure and social activities are reduced. In [19], an evaluation of the early effects of curfews in France during the second wave is done, analyzing the different variables and proposing improvements to increase the yield of the measures.
- Travel limitations: travel to and from hot spots has been limited, imposing measures such as full travel bans, or the need for quarantine after the trip. In [20], the effects of travel bans are studied, concluding that their main effect is the delay in propagation, but not the prevention of spreading.

These measures have proven effective to several degrees. In general, they help reduce the speed of spreading [20], relieving the pressure on hospitals and Intensive Care Units (ICUs). For instance, Reference [21] compares the social distancing measures enforced in Denmark and Norway with the less restrictive Swedish approach, concluding that there is a clear correlation between proximity and the number of hospitalizations and ICU pressure. Nevertheless, none of the aforementioned measures have stopped the pandemic. These policies also come at a great economic cost, as detailed in [22], which, while considered secondary given the risk to citizens’ lives, is not an unimportant issue.

For this reason, several alternative and/or complementary technology-based solutions have been proposed. Contact tracing apps showed great potential for monitoring the spread of the virus and warning potentially exposed individuals early on, before they could become a prolific spreading vector. Reference [23] reviews the experience in different countries with contact tracing apps, showing how in South Korea, Vietnam, Japan, and Taiwan, they helped to reduce the propagation, while the implantation of this technology failed in the western countries, such as the USA, the EU countries and UK, due partially to implementation failures (both in the software and the support infrastructure) and mistrust in public authorities.

## 3. Location Technologies

In the last years, with the advent of mobile communications, location-aware services [24] and network management [25] have grown in importance. The problem of location consists in obtaining the coordinates of a user; mainly their latitude and longitude. The altitude can be obtained from the former coordinates using elevation maps, assuming that the user is on the ground.

**Figure 1:**
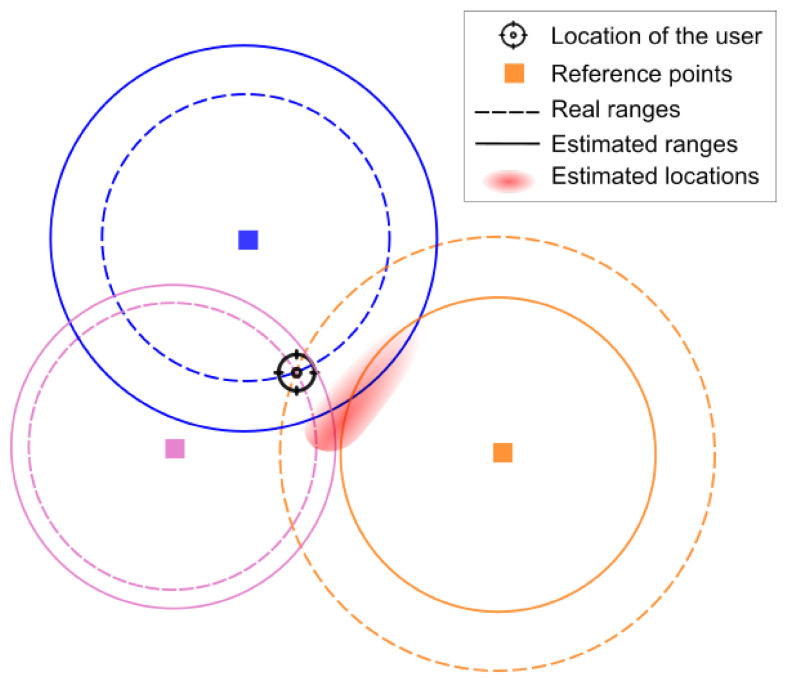
Location using trilateration.

To find the location of the user, one of the most common techniques is trilateration [26], which uses at least three reference points, with a higher number of references being used for increased precision if available. As shown in Figure 1, the distance (or *range*) from the subject to each reference point defines a circle where the subject could be located. The dashed lines represent the ranges for each reference point. The point where the three circles cross is the actual location. In practice, only an estimation of the ranges can be obtained (represented by the full line circles), adding some error to the real range. This gives rise to three circles that do not cross at one point, creating uncertainty in the estimated location, represented in the figure as the red smudge. A higher error in the range estimation will be transformed into a higher error in the location. To obtain a specific point within the smudge, techniques such as Least Square optimization [27] may be used.

Indoor and outdoor locations have different use-cases and challenges. Normally, indoor scenarios are more challenging for radio-based range estimation techniques [28], because of reflections and Non-Line-of-Sight (NLOS) propagation. In NLOS, there are obstacles, such as walls or furniture, between the transmitter and receiver. Since the radio waves may travel a longer distance to get around these obstacles, the estimated ranges will also increase. Outdoors, Line-of-Sight (LOS) communications are more common, leading to potentially more precise range estimations. On the other hand, the sizes of features (streets, lanes, etc) outdoors are bigger than those indoors (rooms, corridors, etc.), so a more precise location is required indoors to better represent the status of the target.

Several technologies are used for location. Some of the most important are the following.

- Global Navigation Satellite System (GNSS): location systems that use a constellation of satellites as reference points for trilateration. They provide an accuracy of a few centimeters with specific techniques [29], but they are limited to outdoors in zones with high sky visibility where LOS is possible. Several GNSS systems are in use: GPS (USA), GLONASS (Russia), BDS (China), and Galileo (EU).
- Cellular Network Location [30]: Cellular networks consist of a deployment of base stations over the geography. At each point in time, a terminal is connected to at least one of these base stations, which already provides approximate information on its location within the coverage area of that base station. Additionally, mobile terminals regularly collect information on the received power of the serving and neighboring base stations to check for potential handovers. These measurements, which are relayed to the network, can be used to estimate the range to each base station for trilateration, achieving a finer precision of around a few hundred meters, as shown in [31]. Novel 5G networks, with denser network deployments, and with the addition of Artificial Intelligence (AI) and Machine Learning (ML) techniques, promise higher precision. Reference [32] reviews both the possibilities for improving network location in 5G and for using location information for enhancing other 5G processes. This focus on location both as an enabler of 5G and a byproduct of the network infrastructure is also the core of the LOCUS project [33]. Normally, cellular location works better in outdoor scenarios, although the emergence of femtocells helps to improve location indoors.
- Ultra-Wideband (UWB) location and WiFi Round-Trip Time (RTT) [34]: in interiors, where GNSS is not effective and NLOS propagation dominates, the cm-level precision is achieved with UWB. This system consists of reference points that receive very short pulses from the devices and answer with another pulse. The RTT is then used by the devices to estimate the range to each reference point. In [35], a comparison between the main commercial UWB positioning systems is done. Recently, IEEE 802.11mc-compliant WiFi devices have used a system based on the same principle and reliant on mesh network deployments.
- WiFi fingerprinting: listing the WiFi access points visible in a specific point in space, will produce a unique combination (or *fingerprint*) of identifiers. In [36], the different fingerprinting-based algorithms for WiFi are reviewed. Trilateration is not used in fingerprinting; in [37], a comparison with WiFi trilateration is done, concluding that fingerprinting achieves a higher precision. This method allows a location accuracy of a few meters, with the condition that the location has been previously scanned and no major changes have occurred in the environment. It works both indoors and outdoors, and its accuracy depends on the quality of the precompiled map of fingerprints.
- Bluetooth: the wide availability of this technology for short-range communications makes it a good candidate for location in interiors. The low range of Bluetooth allows a high location precision in dense Bluetooth deployments [38]. The use of ranging techniques based on Bluetooth has also been proposed [39].
- Magnetic-field-based location: these systems use the local variations in a magnetic field to determine the location of the user. The approach is similar to WiFi fingerprinting, although the measurements are much more sensitive to sensor manufacturer variations, requiring specific approaches to this problem [40,41].
- Dead-Reckoning: sensors such as accelerometers and compasses can be used to estimate the speed and heading of the target and compute its trajectory. This method is used, for instance, for calculating the trajectory of pedestrians [42,43]. To avoid cumulative errors, these techniques are combined with some of the other methods.

These technologies can also be fused to improve the location accuracy [44] and availability [45], using ML and AI to combine the advantages of each source.

Out of these techniques, only cellular network location can be used for mass tracking of the population, since its only requirement is that the subjects carry a connected terminal. Such terminal does not need any special equipment, software, or configuration; location relies solely on necessary parts of the protocol. It is up to the network operator to enable mass location and share the information with public authorities. Nevertheless, these data are not shared very often by operators, because of industrial confidentiality, privacy concerns (further discussed in Section 4), and in some cases the high price tag of the datasets. As a consequence, there are not many studies with real examples of mass tracking. Over the years, since cellular networks rolled out, a limited number of geographical studies have been able to exploit scarce datasets for better understanding of certain aspects of human mobility. For instance, in [46], mobile phone data are used to estimate the mobility of users after the 2010 earthquake in Haiti, concluding that it would help to better coordinate the relief efforts. In [47], a method for studying mobility patterns of anonymous individuals is presented, studying the *geo-social radius*, which indicates the most likely location of an individual. In [48], the data are used to study regional mobility patterns in Ireland. Individual mobility is also studied in [49] (Boston) and [50] (Beijing), where the focus is set in urban mobility. In [51], mobile data are used to estimate the population density and its seasonal variations of different municipalities in Portugal and France. In [52], the analysis is focused on the population mobility between two geographical areas (specifically on the border between Estonia and Finland). These studies do not tackle the technical issues of mass tracking, which are discussed in [53], with a focus on scaling up the required calculations and reducing the computational load. More recently, mass tracking has been proposed for the COVID-19 pandemic; for instance, in [54], the impact of the lockdown in France over the mobility of citizens is studied using mobile phone data, and in [55], the same study is done for Shenzhen (China). In [56], the effects of the regional mobility restrictions in Italy are evaluated using mobility data.

## 4. Privacy Concerns

The fact that the operator can enable massive location monitoring without the need of the users having to perform any action has some problematic implications. The main one is that location monitoring can be done without the explicit consent of the users, violating their privacy. A survey on the main privacy issues and proposed solutions is done in [57].

The storage of the location history of users is a liability for mobile network operators. A security breach could result in a leak in the information, which could subsequently be used for malicious purposes. These leaks may come not only from badly protected databases but also from the core network design elements; for instance, in [58], the researchers used the common channels in the GSM radio stack to identify and track the movements of users with off-the-shelf components.

In a more pessimistic scenario, there are concerns that mass tracking of citizens could be used by hostile public authorities to enable a “Big Brother” scenario. In [59], the legal boundaries of surveillance systems for employees are explored. While the law may offer a certain degree of protection in the private sector, the sole existence of the possibility of such surveillance may result in abuse by public authorities, as described in [60]. In [61], further legal and ethical aspects of the current use of location technologies in the COVID-19 pandemic are reviewed, and the need for a surveillance decommissioning phase after the crisis is finished is outlined.

The main solution to these concerns comes in the form of anonymization. To keep the relevant information (e.g., population flow among geographical units) without risking the privacy of the users, all the information that can lead to the identity of the user is removed or obfuscated. Even anonymized or obfuscated user or terminal information is not enough for privacy preservation; for instance, in [62], location data are de-anonymized exposing home addresses of individuals. Persons tend to follow certain movement patterns every day, in such ways that individuals can be singled out by them, as has been demonstrated even in generalist press studies [63]. In [64], an attempt at improving the quality of anonymization by obfuscating identifiers frequently is proposed. Another solution is aggregation [65]; instead of storing and providing data on individual users, aggregates for the relevant magnitudes are calculated. Properly aggregated data is the only privacy-preserving solution that works if the number of individuals in each cluster is kept adequately high to blur individual patterns. This is, for instance, the approach used in the Google COVID-19 data [66]. As a complementary solution, laws such as the European Unions’ General Data Protection Regulation (GPDR) Article 17 [67] forces companies to erase personal information under the request of the users.

## 5. Pandemics Monitoring with Cellular Networks

This Section explores several ways that cellular network location can be used for monitoring mobility in the context of a pandemic.

### 5.1. Geographical Unit Mobility Monitoring

Having a clear picture of the mobility between geographical units may help monitor the effectiveness, degree of non-compliance, etc., of the limitation measures described in Section 2. It may also be used as a tool for predicting new hotspots.

**Figure 2:**
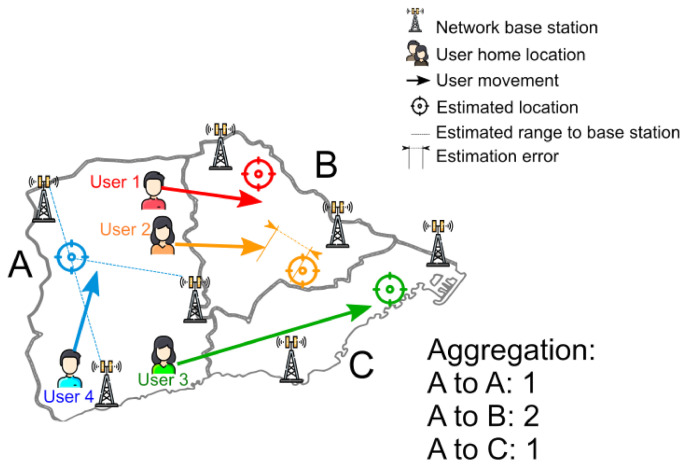
Measurement of mobility among geographical units.

To measure mobility, a *home* and a *destination* unit are assigned to each user. The home unit is established either with the home address of the user, or as the unit where the user spends a predetermined proportion of nights. The destination unit is where the estimated location of the user is at each interval of time. Different strategies can be used to determine the destination if the user moves during the time interval, for instance, counting only the area where the user spends most of the time or counting both as the destination. The flow of population between two units is calculated by either counting the number of users with the same home unit that are localized in a different unit at a given time or counting the number of users that moved from one cell to another in consecutive time intervals. In Figure 2, there are four users whose home cell is *Cell A*. These users move to other cells (except *User 4*), where their locations are estimated by the network base stations. For *User 4*, the estimated ranges used for trilateration are represented (dashed lines). These ranges are based on the received power reported by the users’ terminal, with metrics such as the Reference Signal Received Power (RSRP) for LTE. A propagation model that relates distance with the RSRP is used to estimate the range. These ranges will have a certain error (since distance is not the only factor that determines the RSRP), which will be translated into a location estimation error (depicted for *User 2*). The user flow algorithm will then aggregate the user movements to determine the traffic between cells. In this figure, the traffic from *Cell A* to *Cell B* is 2 (*User 1* and *User 2*), to *Cell C* is 1, and to itself is 1. Some studies that rely on a similar analysis are the aforementioned [52], as well as [68,69], both of which use anonymized roaming information to determine the status of tourism in a region.

### 5.2. Real Time Crowd Monitoring

As shown in Section 2, the COVID-19 virus spreads easily in crowds. Having a real-time picture of where people are crowding may help citizens avoid going into crowds and authorities to prevent the build-up of such crowds.

Coincidentally, crowd monitoring is also relevant for cellular networks. The effects are registered in the performance metrics (*Key Performance Indicators*, KPIs) of the nearby base stations in the network monitoring system. Usually, an abnormally high number of registered devices or offered traffic reflected in the *Number of Active Terminals* and *Number of Connection Attempts* KPIs, respectively, is a clear indicator of crowds. These situations may produce service degradation when the traffic is above the capacity of the network, affecting other KPIs such as the *Accessibility*, which reflects the proportion of successful connection attempts to the network.

The monitoring of traffic can be used for determining that a certain area served by a base station is crowded. In city centers, where the base station deployments are denser, the precision is higher. This solution is completely privacy-preserving since KPIs are aggregated by definition and have no personal or terminal device identifier.

As stated earlier, crowd monitoring is very relevant to the operation of a mobile network. Therefore, it is an area where much research has been done. For instance, in [70], crowd monitoring and prediction are used for activating small cells, that is, additional cells that can help relieve the high network load in specific areas. A similar approach is used in [71]. In [72], mobile phone data are used to estimate traffic jams. In [73], performance indicators in a network are explored to determine how crowded certain cells in a mobile network are, and monitor their mobility with neighboring cells, to assess the risk of COVID-19 spread.

### 5.3. Cellular Contact Tracing

The main factor for contagion in COVID-19 is proximity to an infected patient or carrier. The patients and carriers may spread the virus before even showing any symptoms or knowing that they carry the disease, exposing other individuals who may be at risk without anyone knowing it. Only when patients and carriers get tested can they notify the potentially new infected individuals. This is a difficult task, since there may be no social relationship between the patient/carrier and the exposed individual when the exposure occurs.

Contact tracing applications help keep track of users that may have come into contact. For each user, they keep an anonymized list of public identifiers of other users that have come into close range for a prolonged time. Users diagnosed with COVID-19 are required to notify the application, which will then notify all users of the list, without identifying the patient. Although this solution is privacy-preserving, it has one major limitation, which is that a high proportion of the population should actively use the application. As commented in Section 2, this is a major limitation for some countries where contact tracing did not work because users did not install the application.

Most of the developed contact tracing applications in the last year are based on Bluetooth [74], which they use to interchange information among users’ devices in a decentralized manner to keep track of proximity. Other solutions that rely on an application deployed in the terminal have been proposed; for instance, in [75], magnetometer information is used to find proximity between two devices, and in [76], GPS readings are used to compute the exposure of users to possible contagion sources.

Cellular location can provide a similar solution that is not dependent on any application previously installed on the terminal. In this case, the network can keep track of every user location, and determine close contact through big data analytics. Users that do not have the application installed can be notified of the potential exposure.

## 6. Materials and Methods

In this section, the data available for the sample use case is described. The mobility and contagion data described in this section are then studied in Section 7.

### 6.1. Scenario

The collected data reflect the situation in the province of Málaga, in the south of Spain. The national lockdown started on 13 March, and, at that time, the number of tests was very scarce, so most of the cases were detected when symptoms were clearly visible.

**Figure 3:**
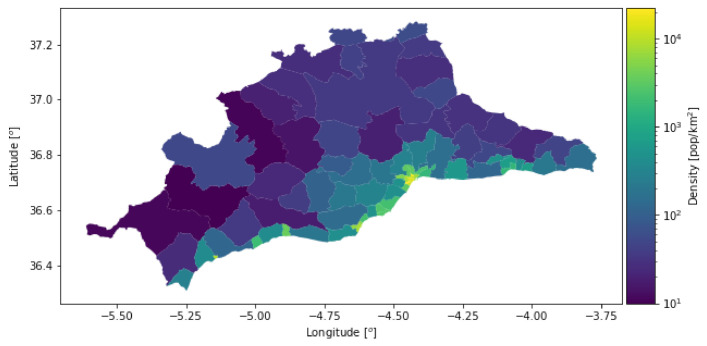
Population density in the province of Málaga.

The province of Málaga has a population of 1.66 million, distributed in 103 municipalities, including the city of Málaga, which comprises 34.58% of the population. Most of the rest of the province’s population is concentrated along the coastline, mainly west of the city of Málaga. The interior of the province is less densely populated. Figure 3 shows the population density of the province. Each subdivision of the map contains at least a population of 5000 people. These subdivisions, or population *cells*, are determined by the National Statistics Institute (INE) [77] of Spain. The province of Málaga contains 80 cells.

### 6.2. Collected Data

There are two separate collected datasets: the mobility dataset and the contagion dataset. The mobility data are collected by the largest national operators [77]. Two different data tables have been collected. In the prior, for each cell, the following fields are collected:

- Resident population of the cell.
- Average number of residents leaving the cell per day.
- Average number of non-residents coming into the cell per day.

In the second table, for each pair of cells, the average number of people moving among them in each direction is collected. The process for determining the movement of users is described in Section 5.1. The data are only given if the traffic is higher than 100, for privacy preservation. The residence of the users is where they spend most of the nights for a period of 60 days, and the destination is where they spend most of the days between 10:00 and 18:00. The data were collected and averaged between 18 November and 21 November 2019. In this paper, these data are used under the assumption that the captured mobility was similar to the mobility in the weeks leading to the confinement in March 2020. This assumption is based on the selection of the collection dates, which are considered to be an average working week without seasonal effects by the *Instituto Nacional de Estadística* (National Statistics Institute) of Spain [77].

**Figure 4:**
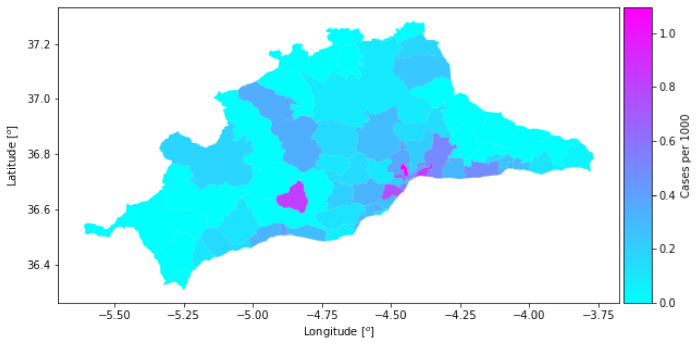
Density of cases in the dataset.

The contagion data collect the total number of detected cases per cell between 3 March and 23 March 2020. The time span covers cases that most likely originated before the confinement (13 March), as a direct consequence of the mobility of previous days captured in the mobility dataset. Figure 4 shows the density of cases per cell.

## 7. Results

In this Section, the datasets presented in Section 6 are analyzed, and the knowledge that can be extracted from them is discussed.

### 7.1. Individual Population Cell Study

In this first analysis, the cells are studied individually. The absolute mobility data (i.e., the number of people moving in or out) per cell are crossed with the contagion data. The initial hypothesis is that the greater the mobility, the higher the probability of contagion.

**Figure 5:**
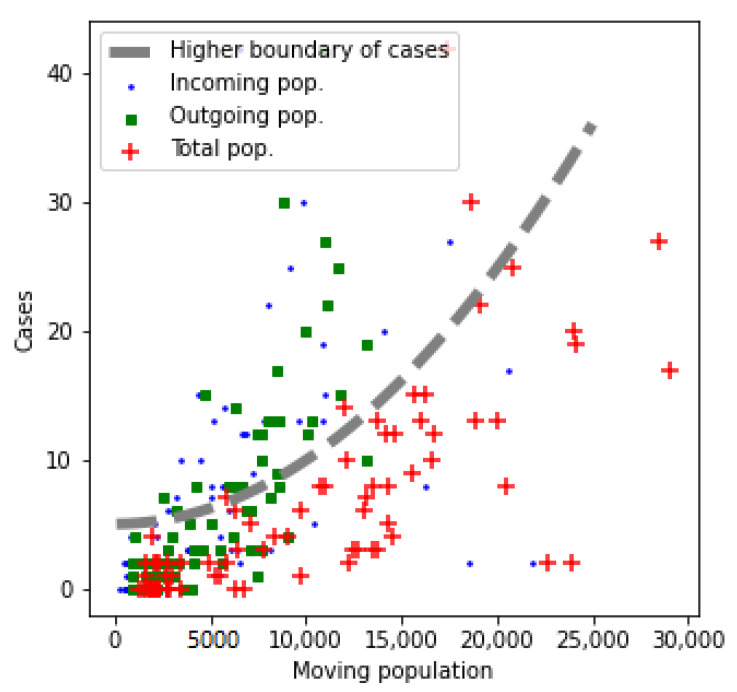
Number of cases vs. mobility.

To verify the hypothesis, the number of cases of each cell was superimposed with the total mobility (Figure 5). In this figure, each point represents one cell, with the horizontal axis showing the mobility, and the vertical axis the number of COVID-19 cases detected in that cell. Three types of mobility can be considered: incoming (i.e., the number of visitors, shown as the blue dots), outgoing (i.e., the number of people leaving a cell, shown as the green squares), and combined (i.e., the total of the previous two, shown as the red crosses).

It can be seen how there is no strong correlation between mobility and cases, so the original hypothesis is not verified. However, it is observed that low mobility seems to guarantee a low rate of infections, conforming a boundary of the maximum number of cases based on mobility, as shown in Figure 5 with the dashed gray line. In other words, the higher the mobility, the higher maximum number of possible cases, although apparently, the number of them depends on other factors.

### 7.2. Mobility Matrix Study

**Figure 6:**
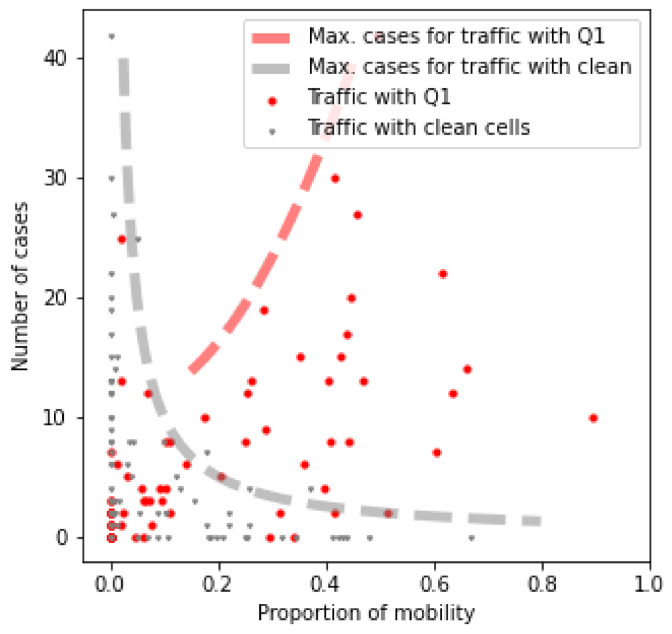
Traffic profiles of all the cases.

In this Section, the analysis of the mobility between cells is discussed. Specifically, the objective of this study is to find if a higher traffic flow with hotspots has any influence on the likelihood that a cell is itself a hotspot. To define the hotspots, the case densities shown in Figure 4 are classified into quartiles. These quartiles correspond to a classification in four categories according to the number of COVID-19 cases: *high incidence* (Q1), *medium-high incidence* (Q2), *medium-low incidence* (Q3), and *low incidence* (Q4). For computing these quartiles, only the cells that had cases are considered, so an additional *no incidence* class groups the rest of the cells. Then, for each cell of the province, a profile counting the flow with cells of each quartile, plus cells with no cases, is obtained. Figure 6 shows the proportions of traffic with the first quartile and with cells with no cases versus the number of cases. The outline of the point cloud for each class is highlighted. The red line represents the higher boundary of infections given the traffic with Q1 cells, and the gray line the higher boundary given the traffic with cells with no cases. From these results, it can be seen that cells with a high traffic proportion with cells with no cases usually have fewer cases. Although a high traffic exchange with hot cells influences the probability of having many cases, they are not the determining factor.

**Figure 7:**
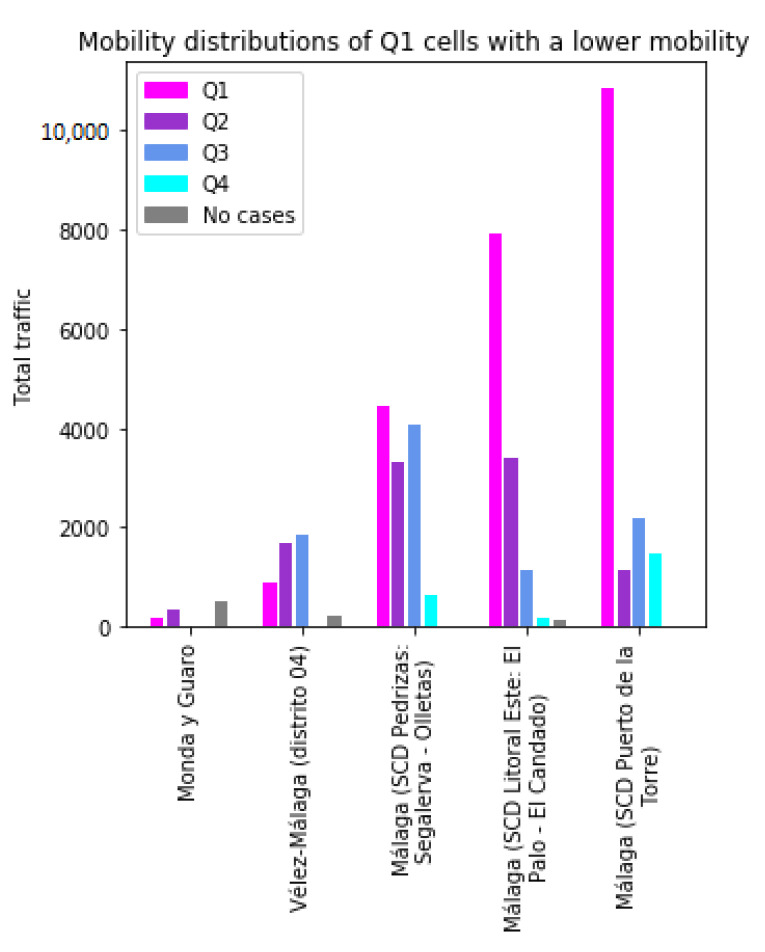
Traffic profiles for anomalous cells of Q1.

To better understand the effects of this classified mobility, the anomalies of Q1 are analyzed. Figure 7 shows the profiles of the five Q1 (*high incidence*) cells with the lowest mobility. These cells would normally have a lower incidence of the virus, due to their low mobility; therefore, some differentiating factors cause them to be in the first quartile. The results show that in three of them, the component of exchange with Q1 cells is very important. The three of them are SCDs of the city of Málaga and correspond to densely populated areas. The mobility may have had an important effect in these cases, increasing the chances that the virus arrived in the cells. An interesting exception is the *Monda y Guaro* cell, which has very low mobility and a low population density. The *Vélez-Málaga (distrito 04)* corresponds to an urban area outside of the city of Málaga. In this case, the mobility is distributed among Q1 to Q3 cells. In these two cells, a high relation with Q1 does not seem to play a central role in their high number of cases, so further investigation would be required.

**Figure 8:**
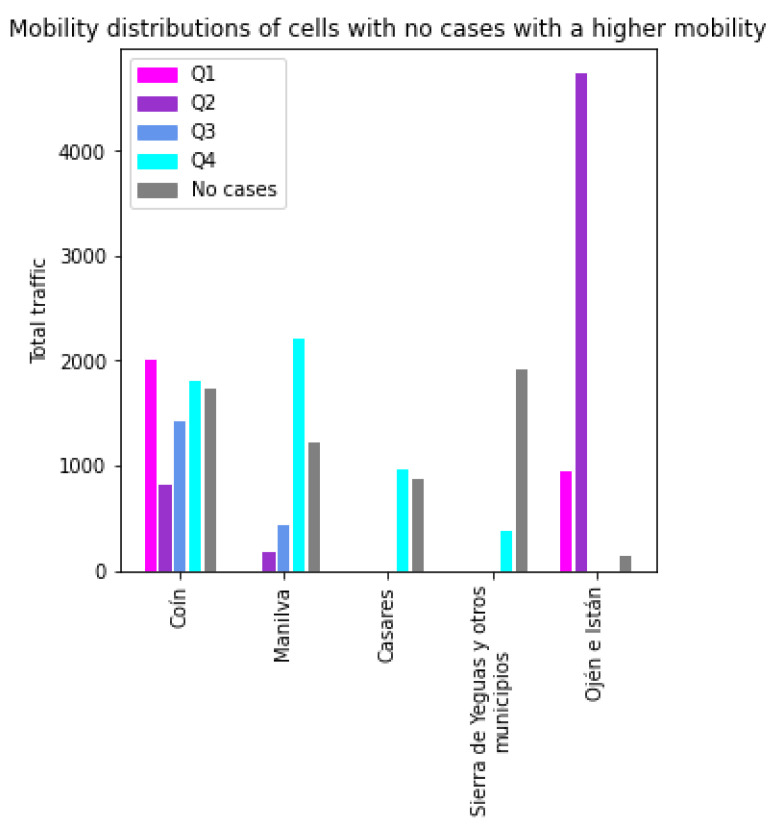
Traffic profiles for anomalous cells of the class with no cases.

Another analysis that will provide valuable information is the study of the opposite cases; that is, cells that have no cases or are in Q4, but with high mobility. Figure 8 shows the traffic profiles of the cells with no cases and the highest traffic. In the case of the *Coín* cell, the mobility is distributed among the five classes. *Ojén e Istán* have high traffic with Q2 cells. The rest of the cells have their mobility concentrated mainly with cells with low incidence. Nevertheless, although these cells are the ones with the highest mobility in their class, their overall mobility is low compared with the cells shown in Figure 7.

**Figure 9:**
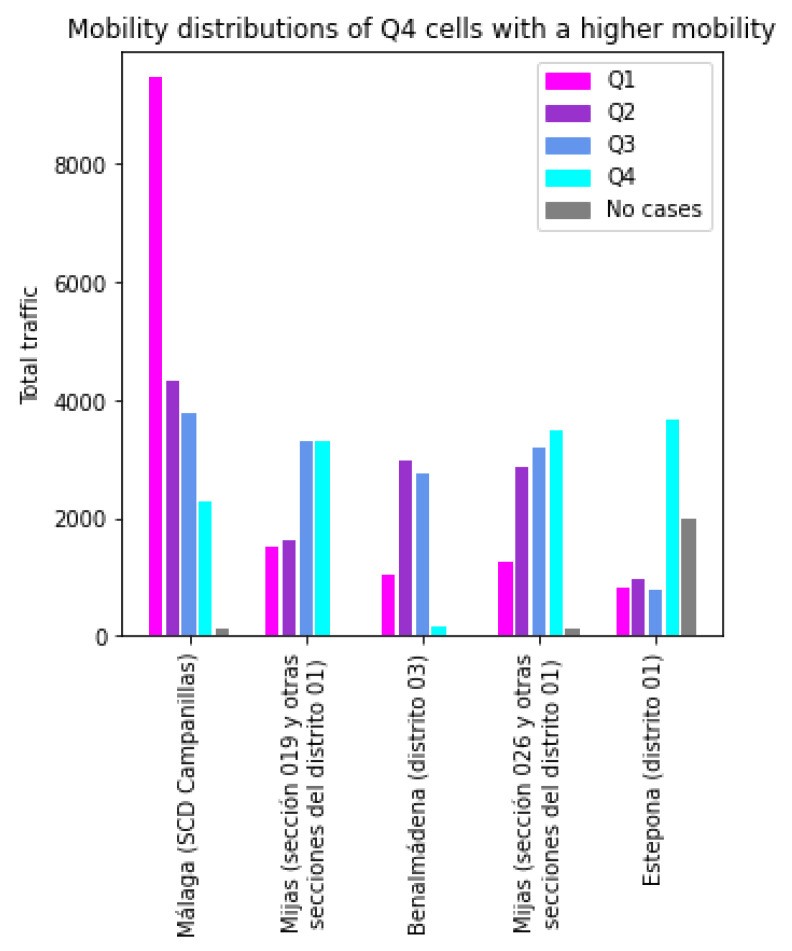
Traffic profiles for anomalous cells of Q4.

In Figure 9, the same study is done for the cells in Q4 (*low incidence*) with the highest mobility. In this case, the aggregated mobility is comparable with the anomalous Q1 cases. The *Málaga (SCD Campanillas)* shows a very high component of traffic with Q1 cells. This means that, while this cell has a high traffic, and that traffic has a very high proportion of interchanges with cells with a high number of cases, it does not affect it negatively. The rest of the cells show lower (but in no case negligible) traffic with Q1 cells. What these results show is that traffic with Q1 does not necessarily imply a higher risk of becoming a hotspot.

The fact that high mobility does not seem to necessarily imply a higher contagion rate may be due to numerous factors. Mainly, the contagion occurs normally in closed spaces and with a certain exposure time. While mobility may increase these encounters in certain geographical areas (for instance, in those that have malls, sports in closed spaces, or residential areas with many shared installations), it will not have any influence in others (e.g., parks or residential areas with dominance of detached houses). Monitoring these encounters would require a combination of cellular contact tracing with the presented geographical unit analysis.

## 8. Conclusions

The COVID-19 pandemic has brought into the spotlight several weaknesses of the globally hyper-connected society of the early 21st century. However, out of this catastrophic situation, several lessons have been learned for the remaining time of this pandemic and for future ones. The most remarkable lesson is that mobility of the population is a key aspect in the spread of a pandemic, and its control, which starts with detailed monitoring, is one of the main measures that can be taken.

To track the mobility of the population, one of the best technologies available nowadays are cellular devices, which most people carry at all times. Location is a fundamental part of cellular networks, so they are a source of information that already performs the tracking that public authorities and individuals need for pandemic spread control and self-protection. This convenience comes at a potential cost of privacy, so legal and technical measurements must be taken to balance the usefulness to public authorities with the protection of the individual, avoiding the scenario of a surveillance instrument imposed with the excuse of the common good.

This paper has reviewed some of the potential applications of mobile network location for pandemic control. Geographical unit mobility monitoring was described as a fundamental asset for helping to decide when and where to impose restrictions and track their enforcement. Real-time crowd monitoring can help find where people are agglomerating, so individuals can avoid the risk of exposure and so public authorities can take the appropriate measures, and cellular contact tracing can be used as an alternative to the existing contact tracing solutions.

Finally, a specific use case for geographical unit mobility monitoring with real data of the province of Málaga (Spain) was shown. The conclusion drawn was that increased mobility increases the higher boundary of possible cases, and that geographical units with high traffic with hotspots have a higher chance of becoming hotspots on their own.

## Data Availability

Mobility dataset available at: https://www.ine.es/experimental/movilidad/experimental_em.htm (accessed on 13 May 2021). Patient data cannot be disclosed due to privacy policy.
